# Different Responses of Soil Bacterial Communities to Nitrogen Addition in Moss Crust

**DOI:** 10.3389/fmicb.2021.665975

**Published:** 2021-09-10

**Authors:** Tingwen Huang, Weiguo Liu, Xi-En Long, Yangyang Jia, Xiyuan Wang, Yinguang Chen

**Affiliations:** ^1^Ministry of Education, Key Laboratory of Oasis Ecology, College of Resources and Environment Science, Xinjiang University, Urumqi, China; ^2^School of Geographic Sciences, Nantong University, Nantong, China; ^3^College of Environmental Science and Engineering, Tongji University, Shanghai, China

**Keywords:** nitrogen deposition, moss crusts, seasonal changes, bacterial composition, community diversity

## Abstract

Bacterial communities in soil serve an important role in controlling terrestrial biogeochemical cycles and ecosystem processes. Increased nitrogen (N) deposition in Northwest China is generating quantifiable changes in many elements of the desert environment, but the impacts of N deposition, as well as seasonal variations, on soil bacterial community composition and structure are poorly understood. We used high-throughput sequencing of bacterial 16S rRNA genes from Gurbantünggüt Desert moss crust soils to study the impacts of N addition on soil bacterial communities in March, May, and November. In November, we discovered that the OTU richness and diversity of soil bacterial community dropped linearly with increasing N input. In November and March, the diversity of the soil bacterial community decreased significantly in the medium-N and high-N treatments. In May, N addition caused a substantial change in the makeup of the soil bacterial composition, while the impacts were far less apparent in November and March. Furthermore, the relative abundance of major bacterial phyla reacted non-linearly to N addition, with high-N additions decreasing the relative richness of *Proteobacteria*, *Bacteroidetes*, and *Acidobacteria* while increasing the relative abundance of *Actinobacteria* and *Chloroflexi*. We also discovered that seasonality, as characterized by changes in soil moisture, pH, SOC, and AK content, had a significant impact on soil bacterial communities. Significant variations in the makeup of the community were discovered at the phylum and genus levels throughout the various months. In May, the variety of soil bacterial community was at its peak. Further investigation showed that the decrease in soil bacterial diversity was mostly attributed to a drop in soil pH. These results indicated that the impact of N deposition on the soil bacterial community was seasonally dependent, suggesting that future research should evaluate more than one sample season at the same time.

## Introduction

Biological soil crusts (BSCs) are common in arid ecosystems. They are made up of mosses, lichens, cyanobacteria, algae, fungus, and bacteria and help to keep the soil wet, prevent erosion, and promote plant development (Belnap and Lange, 2001). Bacteria make up the majority of BSCs and play an important role in their development and persistence (Wang et al., 2015). Meanwhile, bacteria have been recognized for their important role in the breakdown of organic materials in the desert environment (Moquin et al., 2012). The quantity, variety, and function of the soil bacterial community, on the other hand, may change in response to external environmental variables such as N deposition (Wang et al., 2018), the dry-wet cycle (Angel and Conrad, 2013), and temperature (Abed et al., 2015). Thus, studying changes in the soil bacterial community may aid in better understanding of the impacts of environmental change on soil ecosystem processes.

Anthropogenic N deposition (mostly nitrogen oxide and ammonia) has more than tripled in the last five decades as a result of agricultural fertilizer and fossil fuel burning (Bobbink et al., 2010; Galloway et al., 2014). The rate of N deposition is expected to rise steadily and may double by 2050 (Phoenix et al., 2012). Empirical research has revealed that N deposition has a significant impact on the bacterial community through plant-soil feedbacks (Li H. et al., 2016; Zeng et al., 2016). For example, N deposition may increase plant production, reduce root development, change bacterial respiratory substrates, and therefore influence bacterial responses to N deposition (LeBauer and Treseder, 2008; Treseder, 2008). Moreover, many studies have shown that the effects of N deposition on soil bacteria are affected by soil characteristics (Jia et al., 2021; Yue et al., 2021). Previous meta-analysis showed that N deposition reduced soil bacterial diversity and the relative abundance of *Actinobacteria* and *Nitrospirae* via lowering soil pH and changing soil nutritional conditions (Wang et al., 2018). Similarly, one recent study found that N addition-induced acidity was the primary driver to the decline in soil bacterial diversity (Yang et al., 2020). Nonetheless, Liu et al. (2020) discovered that the response of bacterial community composition and diversity to N deposition had a N concentration threshold, beyond which greater N concentration inhibited bacterial diversity. DeForest et al. (2004), on the other hand, found that increased N deposition had no impact on the abundance or composition of bacteria in a hardwood forest. These inconsistencies suggested that the reactions of the soil bacterial community to N deposition are still unknown and need more investigation.

Understanding the dynamics of the desert soil bacterial population is critical for long-term desert management and accurate forecasting of desert ecosystem conditions in the face of future environmental change scenarios (Cregger et al., 2012; Jia et al., 2021). Soil bacterial communities are known to be influenced by seasonal changes in soil moisture and temperature (Liu et al., 2018; Wang et al., 2020). The organization of bacterial communities in dryland ecosystems were shown to be strongly related to soil moisture content (Angel and Conrad, 2013; Wu et al., 2020; Jia et al., 2021). Furthermore, the soil bacterial community has undergone substantial seasonal variations as a result of temperature (Abed et al., 2015). During warm times, the soil bacterial community richness index (ACE and Chao1) and diversity index (Shannon) values were the greatest (i.e., summer; Jia et al., 2020).

Moss crust, which is common in the Gurbantünggüt Desert, is considered the most mature crust type (Belnap and Lange, 2001). Moss crusts, on the other hand, are very susceptible to environmental changes owing to their basic structure and absence of a cuticular barrier (Juha et al., 2015). In recent decades, the Gurbantünggüt Desert has seen increased N deposition, as well as significant seasonal fluctuations in soil temperature and moisture (Li et al., 2014). Although the effects of N deposition on soil bacterial communities dominated by lichens or cyanobacteria have been studied fairly thoroughly (Mueller et al., 2015; Wang et al., 2015), the effects of N deposition in combination with seasonal changes on soil bacterial communities in moss crusts remain unknown. Thus, we examined the soil bacterial community in moss crust, Gurbantünggüt Desert, in a controlled experiment plot that received N addition at four different levels (0, 1.8, 3.6, and 7.2 g N m^–2^ year^–1^). The effects of various amounts of N on the community composition and diversity of moss crust soil bacteria throughout seasons were investigated in this research. We predicted that (1) N addition would reduce soil bacterial community diversity and alter bacterial community composition, and (2) N addition’s impacts would be seasonal, with less influence in May when circumstances are hot and dry.

## Materials and Methods

### Sample Sites

A simulated N deposition experiment was carried out in the southern edge of the Gurbantünggüt Desert (44°11’–46°20’N, 84°31’–91°20’E), China’s biggest permanent and semi-fixed desert (4.88 × 10^4^ km^2^), situated in the Jungger Basin, Xinjiang, at a height of 400–600 m. The climate in the area is characteristic of a continental dry environment, with an annual average temperature of 6.6°C and a maximum temperature of more than 40°C. The annual mean precipitation is 70–150 mm, with the most of it falling in the spring, while the yearly evaporation is 3,000–3,500 mm. In the winter, there is a thick snow cover of more than 20 cm, and the snow cover is constant for approximately 95–110 days (Hui et al., 2016). The desert contains a variety of semi-fixed sand dunes ranging in height from 10 to 50 meters and lengths ranging from tens to hundreds of kilometers. The primary vegetation consists of four shrub species, *Haloxylon ammodendron*, *Haloxylon persicum*, *Ephedra distachya*, and *Ceratoides latens*, interspersed with patches of well-developed BSCs, and the moss crusts are mostly found on the inter-dunes, plant interspace, and underplant canopies.

### Experimental Design

Twelve experimental plots were constructed across a 100 × 100 m area based on the distribution of moss crusts over interdunes. Each plot (5 × 5 m) was randomly assigned one of four N levels. Each plot was surrounded by a 25 cm stainless steel frame, 20 cm of which was buried in the soil. External environmental variables were eliminated by removing any interfering plants from the plots, and the dead branches and debris covering the plots and adjacent regions were cleaned on a regular basis. Considering the background level (3.6 g N m^–2^ year^–1^) of atmospheric N deposition in Xinjiang over the last 10 years (Huang et al., 2015), N addition was applied at four levels in our experiment: control (N0, 0 g N m^–2^ year^–1^), low-N (LN, 1.8 g N m^–2^ year^–1^), medium-N (MN, 3.6 g N m^–2^ year^–1^), and high-N (HN, 7.2 g N m^–2^ year^–1^), and each N treatment had three replicate plots. At the end of March 2017, NH_4_NO_3_ was diluted in 1 L of deionized water (equal to 1 mm precipitation) and uniformly sprayed over the LN, MN, and HN plots in each block. Each control (N0) plot received 1 L of deionized water. We performed the N addition in March because agricultural activity in the adjacent study area peaks in March, when a significant quantity of N fertilizer is utilized, potentially increasing N emission and deposition on desert lands (Zhou et al., 2012). Furthermore, by March, the snow in the study region had almost entirely melted.

### Soil Sample Collection

We collected soil from the research region on the 25th of May 2017 (May, the short-lived plant growth season), the 10th of November 2017 (November, the soil freezing time), and the 16th of March 2018 (March, the snowmelt period). Before taking the cores, the moss crust layer was removed from the soil surface. Using a sterile trowel, five soil cores (with a depth of 5 cm and a diameter of 3.5 cm) were collected from five random locations on each plot. To reduce geographic variability, the five soil cores were combined into a single sample. Finally, we collected 36 soil samples (4 N levels × 3 repetitions × 3 months). Soil samples were placed into a portable chilled box and transferred to the laboratory after plant roots and big stones were removed using a 2 mm screen. Each sample was then subdivided into two sub-samples, one of which was kept at −80°C and used to extract soil DNA and gain information on bacterial community structure, and the other of which was air-dried to assess soil physicochemical characteristics.

### Physicochemical Analysis of Soil Samples

Soil organic carbon (SOC) was estimated by using the potassium dichromate-sulfuric acid oxidation method; total nitrogen (TN) was determined following Kjeldahl digestion on a Nitrogen Analyzer System (KJELTEC 2300 AUTO SYSTEM II, Foss Tecator AB, Höganäs, Sweden). Available nitrogen (AN) was estimated by the alkali-hydrolysis diffusion method. Total phosphorus (TP) was determined by the H_2_SO_4_-HClO_4_ digestion method. Available phosphorus (AP) data was determined by the NaHCO_3_ extraction molybdenum-antimony colorimetric method. Soil samples were brought to the laboratory and mixed with water at a ratio of 1:2.5 to measure the pH using a compound electrode. Soil NO_3_^–^-N content was extracted with 2 M KCl using a continuous flow analytical system (Santt System; Skalar, Holland) at the Xinjiang University of China. Soil moisture content was measured by drying soils at 105°C for 24 h. Available potassium (AK) was extracted with 1.0 mol/L NH_4_Ac and determined by flame photometry.

### DNA Extraction From Soil Samples and Quantitative PCR Analysis

Total genomic DNA was extracted from 0.5 g soil samples using the TIANamp Soil DNA Kit (TIANGEN BioTech (Beijing) Co., Ltd) following the manufacturer’s instructions. The concentration and purity of DNA were monitored on 1% agarose gels. The DNA concentration was adjusted to 1 ng/μL using sterile water. The V3-V4 region of the bacterial 16S rRNA gene was amplified by PCR (95°C for 1 min, followed by 30 cycles of 95°C for 10 s, 50°C for 30 s, 72°C for 30 s, and a final extension cycle at 72°C for 5 min), using the 341F primers 5’-GCTAYGGGRBGCASCAG-3’ and 806R 5’-GGACTACNNGGGTATCTAAT-3’ (Xia et al., 2015), each sample former primer contains a unique 6 bp Barcode (Bensky et al., 1993). PCR reactions were performed in a 50 μL mixture containing 10 μL of the target DNA extract, 15 μL of Phusion Master Mix (2×, New England Biolabs), 2 μL of sterile water, 3 μL of primer, 0.2 μM of forward and reverse primers, and about 10 ng template DNA. PCR products were detected by 2% agarose gel electrophoresis and target DNA bands were purified using a GeneJET Gel Extraction Kit (Thermo Scientific).

### Library Preparation and Sequencing

Sequencing libraries were implemented using the Illumina TruSeq DNA PCR-Free Library Prep Kit (Illumina, United States) according to the manufacturer’s protocols and index codes were added. Library quality was quantified and monitored on the Qubit@ 2.0 Fluorometer (Thermo Scientific, Waltham, MA, United States) and Agilent Bio analyzer 2100 system (Agilent Technologies, Santa Clara, CA, United States), and then the library was sequenced on an Illumina HiSeq platform (Novogene, Beijing, China). The raw FASTQ files have been deposited in the SRA of the NCBI database under the SRA accession: PRJNA701371.

Raw FASTQ files were demultiplexed by QIIME (QIIME 1.9.1), quality-filtered by Trimmomatic, and merged by FLASH (v1.2.7^1^, Magoč and Salzberg, 2011) with the following criteria: (i) the 250 bp reads were truncated at any site receiving an average quality score of <20 over a 10 bp sliding window, and the truncated reads <50 bp were removed. (ii) exact barcode matching, reads containing ambiguous characters, and more than two base mismatch in primer matching were discarded. (iii) only overlapping sequences >10 bp were assembled according to their overlapped sequence, the reads that could not be assembled were discarded (Caporaso et al., 2010). (iv) the sequences were assigned to each sample according to the primers and 6-bp barcode. Then, the barcodes were trimmed and the sequence direction was flipped.

Operational taxonomic units (OTUs) were clustered at a 97% similarity level cutoff using UPARSE (version 7.1^2^), and chimeric sequences were identified and removed using UCHIME (Edgar et al., 2011). The representative sequences were selected for each OTU and use the Ribosomal Database Project (RDP) classifier^3^ to annotate taxonomic information for each representative sequence, and the confidence threshold was set to 80% (Wang et al., 2007). The sampling depth for rarefaction curve analysis was optimized to 13,025 sequences for soil bacterial. Alpha biodiversity was evaluated using the abundance-based indices of OTU richness, Pielou index, and Shannon index, calculated in QIIME2, to analyze the complexity of bacterial diversity in each sample.

### Data Analysis

One-way analysis of variance (ANOVA) was used to analyze the effect of N addition on soil parameters and bacterial community. Two-way ANOVA was used to determine the effects of N addition and seasonal change on soil environmental parameters and bacterial community. Heatmap of the dominant phyla (or genera) and soil physicochemical properties in each sample were drawn using the R packages (pheatmap, psych, reshape2). Principal coordinate analysis (PCoA), based on Bray-Curtis distances of the OTU level data, was performed to interpret the relative similarity of the bacterial community from each sample. Redundancy analysis (RDA; *post hoc* permutation tests with 999 permutations) was used as the statistical criterion to assess multivariate changes in bacterial community composition among treatments using the “vegan” package in R version 4. 0. 2, the most discriminating environmental factors were selected by the “forward selection” of ordiR2step. Additionally, structure equation models (SEMs) were used to explain how N addition and the seasonal change influences soil bacterial community composition/diversity through hypothetical pathways, the response variables for diversity and composition are Shannon diversity index and community OTUs, respectively. The response variables for soil bacterial community were indicated by principal component scores (both PC1 scores were used) (Wu et al., 2019), which are usually used in SEM analyses. The SEMs were analyzed using IBM SPSS Amos 24, and Adobe Illustrator CC 2019 was used to process the graphics.

## Results

### Effects of N Addition and Seasonal Changes on Soil Properties

**FIGURE 1:**
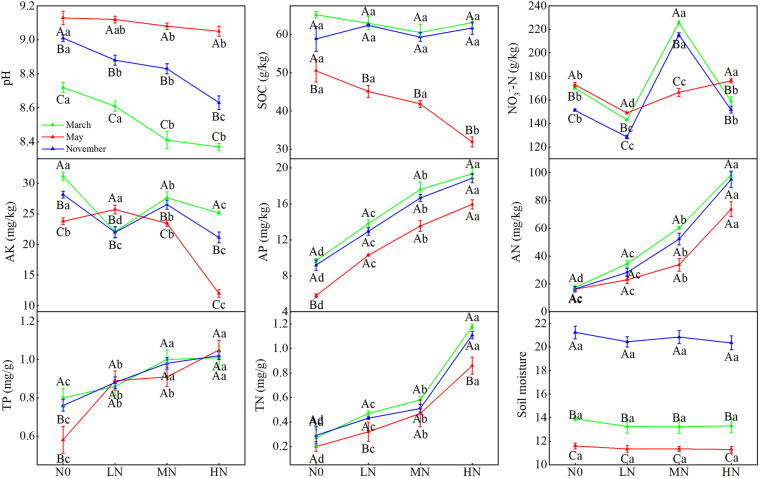
Seasonal changes in soil physicochemical properties in moss crust soil with different levels of N additions. N0 = 0 g N m^– 2^ year^– 1^; LN = 1.8 g N m^– 2^ year^– 1^; MN = 3.6 g N m^– 2^ year^– 1^; HN = 7.2 g N m^– 2^ year^– 1^. Different capital letters indicate a significant difference among the three sampling months with the same concentration; different lowercase letters indicate a significant difference among four N treatments in the same sampling month. Vertical bars show the standard error (SE) (*n* = 3).

Soil AP, AN, TP, and TN content showed increasing trends with N addition, but pH showed a decreasing trend with N addition (Figure 1). Soil NO_3_^–^-N concentrations and AK content both showed significant variations between N concentrations (*P* < 0.05). In May, SOC content dropped linearly across the gradient of N inputs. Simultaneously, we discovered substantial changes in soil pH, moisture, and SOC concentration between sample months (Figure 1; Table 1). In May, SOC content was lowest, whereas soil pH was greatest (*P* < 0.05). Soil moisture was greatest in November (21%), followed by March (13%) and May (11%, *P* < 0.05). Furthermore, we also found significant interactions between N addition and seasonal changes in soil pH, TP, AN, SOC, NO_3_^–^-N, and AK (Table 1).

**TABLE 1:** Effects of nitrogen addition, seasons, and their interaction on soil variables, as indicated by two-way ANOVA statistics.

**Factors**	**Nitrogen**		**Season**		**Nitrogen × Season**	
	**F**	**P**	**F**	**P**	**F**	**P**
pH	0.482	0.037	18.328	0.001	13.121	0.001
Soil moisture	0.010	0.999	972.799	<0.001	5.370	0.056
TP (mg/g)	13.184	0.002	0.210	0.815	71.890	0.004
TN (mg/g)	36.194	<0.001	0.235	0.795	3.299	0.061
AN (mg/kg)	30.356	<0.001	0.260	0.776	5.060	0.020
AP (mg/kg)	14.094	0.001	0.842	0.462	0.058	0.999
SOC (g/kg)	0.153	0.925	22.497	<0.001	3.529	0.038
NO_3_^–^-N (g/kg)	5.669	0.022	0.180	0.838	11.639	0.009
AK (g/kg)	2.259	0.159	1.289	0.322	9.193	0.023

*Values represent mean with standard error in parenthesis. TP, total phosphorus; TN, total nitrogen; AN, available nitrogen; AP, available phosphorus; SOC, soil organic carbon, NO_3_^–^-N, nitrate nitrogen; AK, available potassium.*

### Responses of Soil Bacterial Community Diversity to N Addition and Seasonal Change

**FIGURE 2:**
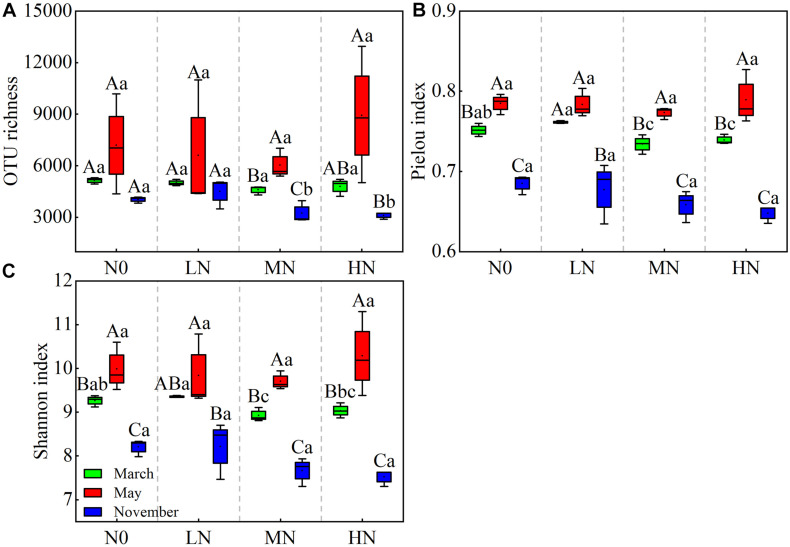
Seasonal changes in soil bacterial OTU richness and diversities in moss crusts with different levels of N additions. OTU richness **(A)**, Pielou index **(B)**, and Shannon index **(C)**. N0 = 0 g N m^– 2^ year^– 1^; LN = 1.8 g N m^– 2^ year^– 1^; MN = 3.6 g N m^– 2^ year^– 1^; HN = 7.2 g N m^– 2^ year^– 1^. Different capital letters indicate a significant difference among the three sampling months with the same concentration of N; different lowercase letters indicate a significant difference among four N treatments in the same sampling month. Vertical bars show the standard error (SE) (*n* = 3).

In May, no significant changes in OTU richness, Shannon, or Pielou index were found as N increased (Figure 2). In November, however, the OUT richness and diversity of bacterial community dropped linearly with increasing N inputs. Simultaneously, MN and HN addition showed a substantial detrimental impact on community diversity in both November and March (*P* < 0.05). Furthermore, the bacterial diversity indices varied between sample months. In May, the soil bacterial community was at its most diverse, with an OTU richness index of 6,029–8,920, a Pielou index of 0.77–0.78, and a Shannon index of 9.70–10.28 (Figure 2; Supplementary Table 1). However, no significant impact was observed in the interaction N treatment seasonal change for bacterial community diversity (Table 2).

**TABLE 2:** Effects of nitrogen addition, season, and their interactions on soil bacterial diversity as indicated by two-way ANOVA statistics, based on the relative abundance of phyla level.

**Factors**	**Nitrogen**		**Season**		**Nitrogen × Season**	
	**F**	**P**	**F**	**P**	**F**	**P**
OTU richness	0.449	0.725	11.634	0.002	0.694	0.627
Shannon index	0.893	0.485	78.595	<0.001	1.230	0.342
Pielou index	1.701	0.243	170.318	<0.001	1.183	0.365
*Proteobacteria*	0.053	0.983	177.079	<0.001	2.384	0.077
*Actinobacteria*	1.784	0.228	124.896	<0.001	6.631	0.001
*Bacteroidetes*	0.828	0.514	29.450	<0.001	1.016	0.450
*Acidobacteria*	3.253	0.081	20.404	<0.001	4.298	0.009
*Chloroflexi*	3.622	0.065	134.703	<0.001	7.034	0.043
*Firmicutes*	0.866	0.497	45.454	<0.001	1.049	0.431
*Cyanobacteria*	2.782	0.110	10.267	0.008	3.250	0.081
*Gemmatimonadetes*	2.956	0.098	70.792	<0.001	1.250	0.333
*Planctomycetes*	1.227	0.362	33.762	<0.001	4.428	0.041
*TM7*	1.807	0.224	124.052	<0.001	3.319	0.078
*Verrucomicrobia*	4.328	0.043	9.887	0.002	1.242	0.318

### Effects of Increased N Addition on Soil Bacterial Community Structure in Different Seasons

**FIGURE 3:**
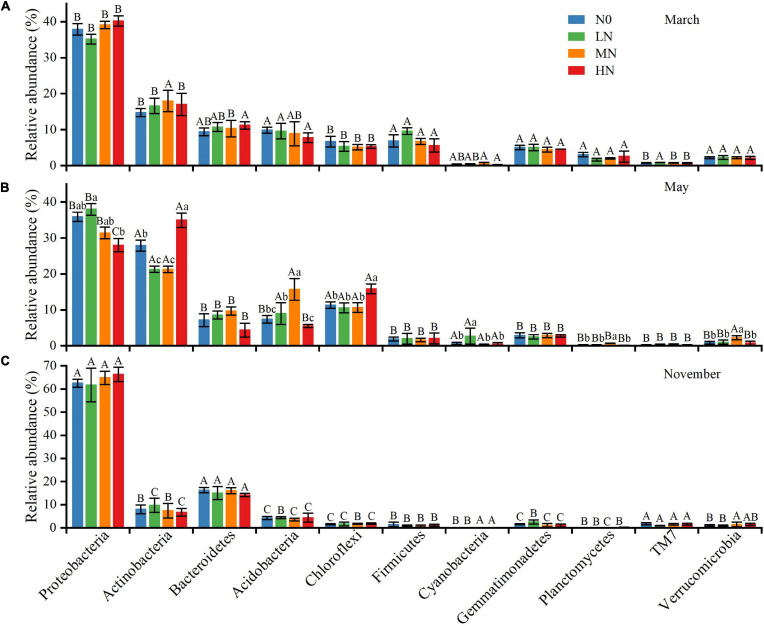
The average relative abundance (%) of bacterial taxa at the phylum level (*n* = 3), March **(A)**, May **(B)**, and November **(C)**. N0 = 0 g N m^– 2^ year^– 1^; LN = 1.8 g N m^– 2^ year^– 1^; MN = 3.6 g N m^– 2^ year^– 1^; HN = 7.2 g N m^– 2^ year^– 1^. Different capital letters indicate a significant difference among the three sampling months; different lowercase letters indicate a significant difference in soil bacterial relative abundance among four N treatments in the same sampling month. Vertical bars show the standard error (SE) (*n* = 3).

*Proteobacteria*, *Actinobacteria*, *Bacteroidetes*, *Acidobacteria*, and *Chloroflexi* were the most prevalent phyla across all N deposition levels and sample months, accounting for 78–93% of total bacterial sequences (Figure 3). Some bacterial phyla were shown to be more susceptible to N addition in May than in November and March (Figure 3). For example, in N-amended soils, the relative abundances of *Actinobacteria* decreased by 6.7 and 6.8% for LN and MN, respectively, but rose by 7.1% for HN. The effects of N addition on the relative abundance of *Acidobacteria*, on the other hand, exhibited the opposite trend. The relative abundance of *Chloroflexi* rose substantially after HN treatment (15.84%). When compared to the N0 treatment, the relative abundance of *Proteobacteria* was higher in the LN (37.97%) and lower in the MN (31.38%) and HN (28.01%) treatments. Furthermore, the five most prevalent genus (>1%) across all N addition treatments were identified (Supplementary Figure 1). The variations in dominating genera were also seen across N treatments. Under N addition, the relative abundances of *Sphingomonas* and *Balneimonas* changed substantially in May and November, whereas *Kaistobacter* changed significantly in all three sample months.

Soil bacteria’s relative abundance changed seasonally. At the phylum level, *Proteobacteria*, *Bacteroidetes*, and *TM7* had the highest relative abundance in November compared to March and May, while *Actinobacteria*, *Acidobacteria*, and *Chloroflexi* had the lowest. *Firmicutes*, *Gemmatimonadetes*, *Planctomycetes*, and *Verrucomicrobia* were more abundant in March than in the previous 2 months. In November, the genera *Sphingomonas*, *Kaistobacter*, *Massilia*, and *Adhaeribacter* had the greatest relative abundance. *Rubrobacter*’s relative abundance was in the sequence of May > March > November. Furthermore, we discovered that N addition and seasonal variation had an interaction impact on the relative abundance of several prominent phyla and genera, including *Actinobacteria*, *Acidobacteria*, *Chloroflexi*, *Planctomycetes*, *Sphingomonas*, *Balneimonas*, *Kaistobacter*, and *Geodermatophilus* (Table 2).

**FIGURE 4:**
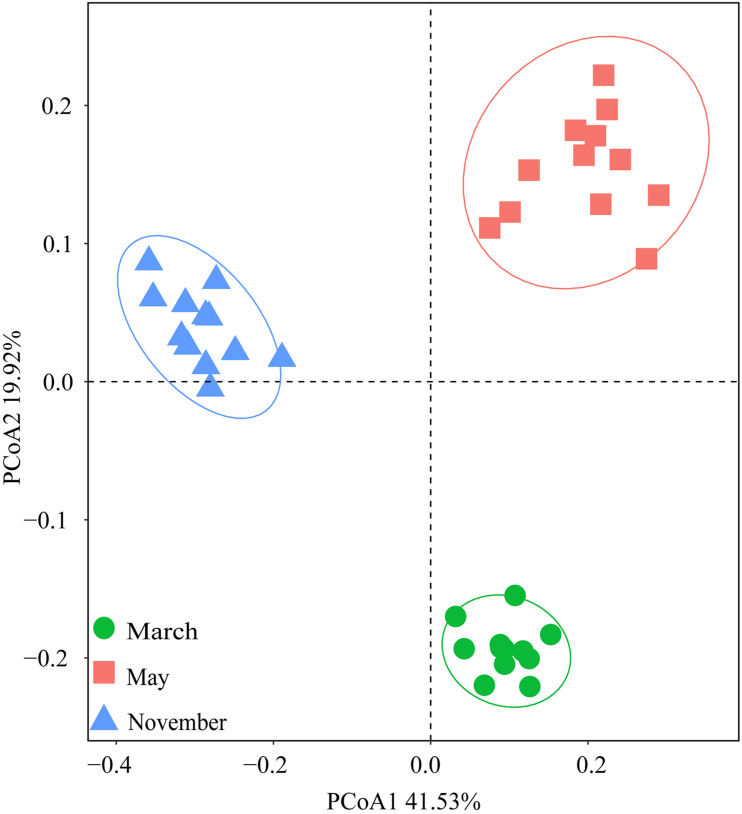
Principal coordinate analysis (PCoA) based on Bray-Curtis distances of soil bacterial community compositions at the OTU level in three sampling months.

Bray-Curtis distances were calculated using Principal coordinate analysis (PCoA) to show the differences in bacterial communities across treatments (Figure 4). PCoAs 1 and 2 explained 41.53 and 19.92% of the total variance, respectively. The bacterial communities were divided based on the months rather than the amounts of N addition. This meant that the impacts of season on soil bacterial composition were greater than the effects of N addition.

### Relationships Between the Bacterial Community Structure and Soil Properties

**FIGURE 5:**
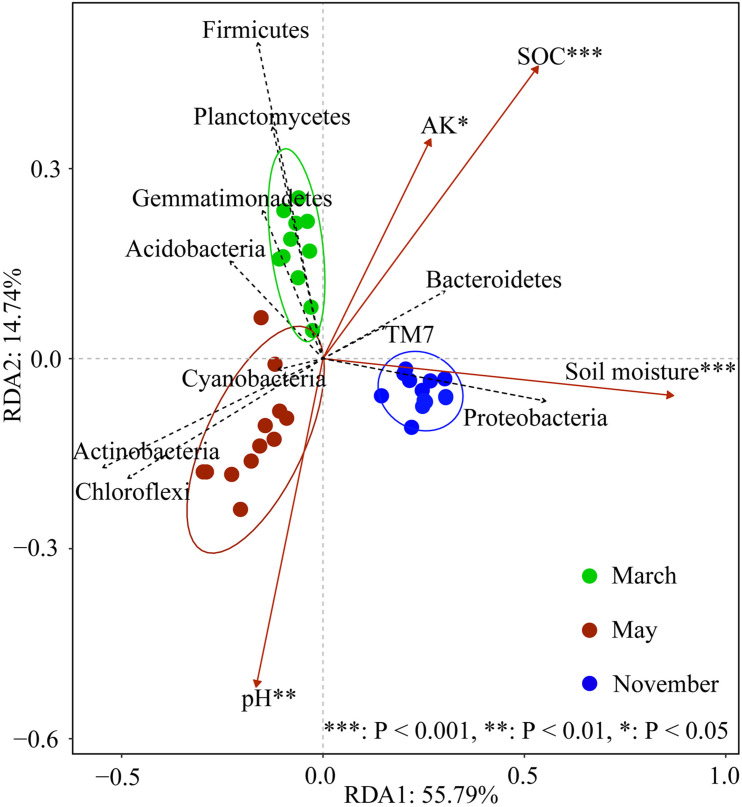
Redundancy analysis (RDA) for the soil bacterial community structure (based on phyla level data, only top 10 phyla are shown in the figure) and environmental variables at different N levels and sampling periods. Only the environmental variables significantly correlated with RDA are shown in the figure. Significance levels are denoted with *p < 0.05, **p < 0.01, and ***p < 0.001.

**FIGURE 6:**
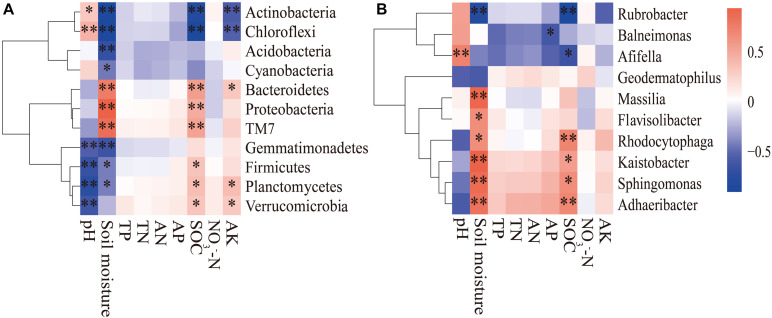
The spearman correlation heatmap of the dominant bacterial phyla **(A)** and genera **(B)** and soil properties. The X and Y axes represent the environmental factors and phyla (or genera), respectively. R in different colors to show, the right side of the legend is the color range of different *R* values. *P < 0.05; **P < 0.01.

The composition of the soil bacterial community was significantly different in different months based on the RDA analysis, which Axis 1 explained 55.79% of the total variance, and Axis 2 explained 14.74% (Figure 5). The result revealed that the soil bacterial community was significantly related to soil moisture (*F* = 49.453, *P* < 0.001), SOC (*F* = 15.17, *P* < 0.001), pH (*F* = 5.162, *P* = 0.007), and AK (*F* = 4.169, *P* = 0.012). Correlation analysis revealed that the bacterial phylum *Actinobacteria* and *Chloroflexi* were positively correlated with soil pH, and negatively correlated with soil moisture, SOC, and AK contents (Figure 6A). Conversely, *Gemmatimonadetes*, *Firmicutes*, *Planctomycetes*, and *Verrucomicrobia* were negatively correlated with soil pH and soil moisture, but positively correlated with soil SOC and AK contents. There was a positive correlation between *Proteobacteria*, *Bacteroidetes*, and *TM7* and soil moisture, SOC, and AK contents. At the genus level, we found that *Afifella* exhibited significant positive correlations to soil pH. *Rubrobacter* exhibited highly negative correlations with soil moisture and SOC content, while *Sphingomonas*, *Adhaeribacter*, *Kaistobacter*, *Rhodocytophaga*, *Massilia*, and *Ramlibacter* correlated positively with soil moisture and SOC content (Figure 6B).

**FIGURE 7:**
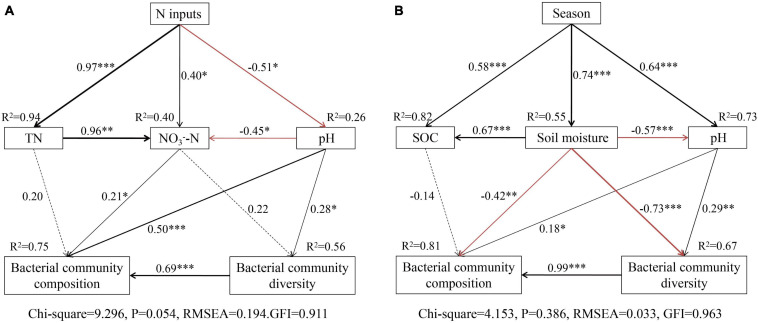
Structure equation model (SEM) analysis of N addition **(A)** and seasonal changes **(B)** on soil bacterial communities via pathways of soil properties. Continuous and dashed arrows represent significant and non-significant relationships, respectively. Adjacent numbers that are labeled in the same direction as the arrow represent path coefficients and the width of the arrow is in proportion to the degree of path coefficients. Black and red arrows indicate positive and negative relationships, respectively. *R*^2^ values indicate the proportion of variance explained by each variable. Significance levels are denoted with *P < 0.01, **P < 0.01, and ***P < 0.001. The low chi-square (CMINDF), non-significant probability level (*P* > 0.05), high goodness-of-fit index (GFI > 0.90), and low root-mean-square errors of approximation (RMSEA) listed below the SEMs indicate that our data matches the hypothetical models.

Structure equation models analysis also showed that N addition significantly affected soil N and pH, and explained 96 and 26% of the total variance, respectively (Figure 7A). Soil pH explained the total variance in the soil bacterial community diversity (56%) and composition (75%) (Figure 7A), while soil N had no directly affected the soil bacterial community, these results shown that increased N mainly affects soil bacterial communities by causing soil acidification. Seasonal changes significantly affected soil moisture, SOC, and pH, and explained 55, 82, and 76% of the total variance (Figure 7B), respectively. Three soil parameters explained 67 and 81% of the total variation in the soil bacterial community diversity and composition. Overall, the bacterial community composition was strongly driven by the soil moisture, pH, SOC, and diversity (Figure 7).

## Discussion

In this research, we discovered that short-term N additions and seasonal variations may have a significant impact on soil physical and chemical characteristics, and therefore affect soil bacterial community diversity and structure. Soil acidification was induced by simulated N inputs, and soil moisture, pH, and SOC varied substantially throughout the three monitoring months. These soil characteristics were strongly linked to changes in the soil bacterial community. Furthermore, the reactions of the soil bacterial community to N deposition were seasonally dependent.

### Effects of N Addition on Soil Bacterial Diversity

In November, the OTU richness and diversity of the soil bacterial community declined linearly with increased N inputs, according to our data. A similar finding was published in the same area, demonstrating that the bacterial diversity of BSCs (i.e., the ACE and Chao1 index) declined linearly with increasing N additions (Wang et al., 2015). Furthermore, our findings revealed that MN and HN addition substantially reduced soil bacterial diversity indices (Shannon and Pielou) in November and March, but had no effect in May. This finding suggested that soil environment variables may influence the impact of N addition on bacterial diversity in the soil. Soil moisture varied considerably across the three study months, with the lowest in May (11%) and the greatest in November (21%). According to Lucas et al. (2011), excessive soil moisture may enhance soil acidification owing to increased metal cation leaching. N addition did definitely reduce soil pH in this alkaline desert soil, particularly in November (0.35 units) and March (0.38 units), which is approximately one unit more than the stated worldwide average of 0.26 units (Tian et al., 2016). According to the correlation study, soil bacterial diversity indices were positively associated with soil pH (*P* < 0.05; Supplementary Table 3). In our research, there were no reactions of soil bacterial diversity to N addition in May, which may be attributed to minor variations in soil pH (0.08 units). Thus, in desert environments, the differential reactions of soil bacteria to N addition may be strongly related to water availability and variations in soil pH.

### Effects of N Addition on Soil Bacterial Community Composition

The composition of soil bacterial communities is closely related to ecosystem functions (Allison et al., 2013). Understanding the mechanisms underlying the effects of environmental change on soil bacterial communities will thus aid in forecasting the response of ecosystem processes to environmental change (Jansson and Hofmockel, 2019). In contrast to our second hypothesis, seven bacterial phyla are significantly affected by N addition in May (Figure 3). At the high N treatment, for example, bacterial assemblages classified as *Proteobacteria*, *Bacteroidetes*, and *Acidobacteria* decreased. Similarly, Li Q. et al., 2016 discovered that high N treatment increased the relative abundance of *Actinobacteria* while decreasing the relative abundance of *Proteobacteria*. *Acidobacteria*, *Alphaproteobacteria*, and *Bacteroidetes* decreased after N enrichment, according to Zeng et al. (2016). Wu et al. (2019) discovered that high N treatment reduced the relative abundance of *Chloroflexi* and *Planctomycetes* while increasing the relative abundance of *Proteobacteria*, *Cyanobacteria*, *Bacteroidetes*, and *Verrucomicrobia*. Wang et al. (2020) discovered that N inputs increased the relative abundance of *Actinobacteria* and *Chloroflexi* while decreasing the relative abundance of *Acidobacteria* and *Verrucomicrobia*, which contradicted our findings. Furthermore, the one-way ANOVA revealed that there was a strong shift in bacterial relative abundance in May, with N addition having a significant effect on seven bacterial phyla (Figure 3B). In November and March, however, no bacterial phylum showed significant changes (Figures 3A,C). This finding suggested that investigating the effects of N addition on the soil bacterial community over a single season could be biased. Several studies have found seasonal dynamics in the abundance and community composition of soil bacterial communities (Liu et al., 2018; Wang et al., 2020). In our study, November and March are typically characterized by low temperatures, whereas May is typically characterized by high temperatures and dry conditions. Buckeridge et al. (2010) found that when soil temperatures are close to 0°C, soil microorganisms and the C and N pool have high stability, which may limit the soil bacterial community’s response to N addition.

The non-linear response of the relative abundance of these bacterial phyla (*Proteobacteria*, *Actinobacteria*, *Acidobacteria*, *Chloroflexi*, *Cyanobacteria*, *Planctomycetes*, *Verrucomicrobia*) to N inputs is perhaps the most intriguing finding in our study (Figure 3B). Previous research has shown that soil bacterial composition is linked to soil pH and nutrient availability (potassium, magnesium, calcium, and SOC; Lauber et al., 2009; Li et al., 2019; Zhang et al., 2020). The RDA findings revealed that soil pH, AK, and SOC concentration all had a substantial impact on the bacterial community, confirming that soil pH, AK, and SOC content were all key variables affecting bacterial structure. Indeed, soil available K content may be an essential regulator on *Proteobacteria* and *Cyanobacteria* abundance shifts, since both altered accordingly with soil available K content variations. In May, however, we discovered that soil pH and SOC concentration dropped linearly with increased N inputs (Figure 1). This finding showed that the shifts of particular bacterial taxonomic groupings could not be anticipated merely by changes in a single soil parameter, and that the complicated response of soil bacterial communities to N addition might be the consequence of a combination of many variables. As Wang et al. (2015), non-linear variations in the relative abundance of *Actinobacteria* and *Chloroflexi* were caused by phased regulation by soil SOC and pH value.

We also found that *Rubrobacter* and *Geodermatophilus* had a greater relative abundance, but their abundances did not vary significantly across various N additions. *Rubrobacter* genera have a general adaptability to hot and dry conditions (Ferreira et al., 1999; Laiz et al., 2009), and certain *Rubrobacter* and *Geodermatophilus* species are known to be extremely radiation-resistant (Yoshinaka et al., 1973; Montero-Calasanz et al., 2015). The features of the two aforementioned genera are critical to the development and persistence of BSCs in dry zones with high UV exposure.

### Effects of Seasonal Changes on Bacterial Community Diversity and Composition

Previous research indicated that seasonal community dynamics existed in soil bacterial communities (Rasche et al., 2011; Matulich and Martiny, 2016). In our research, the most diverse soil bacterial community was found in May, followed by March and November. Meanwhile, we discovered that the number of bacterial phyla and genera with relative abundance (more than 1%) was essentially constant throughout seasonal fluctuations, and the bacteria phyla in May were more susceptible to N addition than the other 2 months. Changes in relative abundance induced by N addition may be the key to May’s greatest bacterial diversity. Furthermore, prior research indicated that seasonal changes in desert soils are driven by variations in soil moisture and temperature, which are known to be significant drivers of changes in soil bacterial communities (Jia et al., 2021). Our findings showed that soil moisture was strongly linked to the variety of bacterial communities studied (Supplementary Table 3). Lan et al. (2018) also found that increasing soil moisture reduced bacterial community diversity, which is consistent with the findings of this research.

Seasonal changes in the composition of soil bacterial communities may be driven by the shifts in climate and resource availability (Firestone, 2006). The soil in our research location, situated in Xinjiang’s Gurbantünggüt Desert, has a relatively high pH and low soil moisture. High pH and low soil moisture were linked to greater evaporation in May when compared to March and November (Yu et al., 2014). Soil moisture, nutrient availability, and pH are examples of environmental factors that may be used to explain the substantial variations in soil microbial community composition between seasons (Smith et al., 2010; Rasche et al., 2011). We discovered that variations in soil nutrient availability may cause the soil bacterial community’s (oligotroph-copiotroph) nutritional strategy to alter. *Proteobacteria* and *Bacteroidetes* are copiotrophic microorganisms with fast growth among the major bacterial groupings (Fierer et al., 2012, 2007). Both of these soil microbial groups were more abundant in March and November, when soil nutrient concentration was greater than in May. Moreover, these bacteria are gram-negative and lack thick cell walls (Fierer et al., 2007; Schimel et al., 2007). To avoid cell rupture, most gram-negative bacteria can quickly dispose of osmolytes upon rewetting, either via respiration, polymerization, or transfer across the cell membrane. As a result, gram negative bacteria may flourish in the time immediately after rewetting (Schimel et al., 2007). Furthermore, the cold-resistant *Bacteroidetes* (*Flexibacter* sp., *Sphingobacterium* sp., *Flectobacillus* sp., *Flavisolibacter* sp.) allow members of this phylum to survive in cold settings (Margesin and Schinner, 1994; An et al., 2010). *Cyanobacteria*, the first group of photosynthetic gram-negative prokaryotes to evolve oxygen, had a greater relative abundance in May (Abed et al., 2010). These results suggest that seasonal variations in bacterial community composition may be ascribed to bacteria’s physiological adaptation to soil temperature, precipitation, and vegetation conditions.

## Conclusion

In November, bacterial OTU richness and diversity declined linearly with increased N inputs, indicating that the impact of N addition on bacterial diversity was seasonally dependent. MN and HN supplementation substantially reduced bacterial diversity indices in November and March (Shannon and Pielou). In May, N addition had a significant impact on the relative abundance of seven bacterium phyla, and the relative abundance of seven bacteria phyla reacted non-linearly to N addition. High N inputs reduced the relative richness of the bacterial phyla *Proteobacteria*, *Bacteroidetes*, and *Acidobacteria* while increasing the number of *Actinobacteria* and *Chloroflexi*. Concurrently, the variety and composition of the soil bacterial community changed substantially across months, with bacterial community diversity indices peaking in May. The composition of the soil bacterial community correlated strongly with soil pH, moisture, SOC, and AK concentrations, suggesting that these factors were important in maintaining the stability of the soil bacterial community. Taken together, the reactions of the soil bacterial community to increasing N vary substantially between seasons, and seasonal variations influenced the soil bacterial community through influencing soil characteristics.

## Data Availability Statement

The datasets presented in this study can be found in online repositories. The names of the repository/repositories and accession number(s) can be found below: https://dataview.ncbi.nlm.nih.gov/object/PRJNA701371?reviewer=92edmcq2pqqj57f9915t3tm9nu.

## Author Contributions

TH: conceptualization, data curation, formal analysis, investigation, software, validation, visualization, and writing – original draft. WL: conceptualization, funding acquisition, investigation, writing, review, and editing. X-EL: data curation, software, writing, review, and editing. YJ and XW: writing, review, and editing. YC: funding acquisition, writing, review, and editing. All authors contributed to the article and approved the submitted version.

## Conflict of Interest

The authors declare that the research was conducted in the absence of any commercial or financial relationships that could be construed as a potential conflict of interest.

## Publisher’s Note

All claims expressed in this article are solely those of the authors and do not necessarily represent those of their affiliated organizations, or those of the publisher, the editors and the reviewers. Any product that may be evaluated in this article, or claim that may be made by its manufacturer, is not guaranteed or endorsed by the publisher.

## Footnotes

1
http://ccb.jhu.edu/software/FLASH/

2
http://drive5.com/uparse/

3
http://rdp.cme.Msu.edu/classifier/classifier.jsp
