# Supplementary material for: Different Responses of Soil Bacterial Communities to Nitrogen Addition in Moss Crust
Source: Front Microbiol. 2021 Sep 10;12:665975. doi: 10.3389/fmicb.2021.665975 (PMC8460773; doi:10.3389/fmicb.2021.665975)
Supplement: Supplementary file 1 [file Data_Sheet_1.zip › Table 1.DOCX]

**Table S1.** A summary of high quality sequence numbers, numbers of operational taxonomic units (OTUs; ≥ 97 % sequence similarity), and diversity estimates obtained from moss crusts soils. To allow comparison of the Number of sequences, OTU richness, Pielou index, and Shannon index, between samples.

| sample period | Sample | Number of sequences | OTU richness | Pielou index | Shannon index |
| --- | --- | --- | --- | --- | --- |
| March | N0 | 39739±1920 | 5142±186 | 0.75±0.01 | 9.26±0.13 |
|  | LN | 40460±3839 | 5004±184 | 0.76±0.01 | 9.35±0.02 |
|  | MN | 40370±2988 | 4596±252 | 0.73±0.01 | 8.92±0.15 |
|  | HN | 37582±1983 | 4797±508 | 0.73±0.01 | 9.03±0.17 |
| May | N0 | 53801±4605 | 7188±2912 | 0.78±0.01 | 9.98±0.55 |
|  | LN | 53624±9002 | 6600±3806 | 0.78±0.02 | 9.83±0.82 |
|  | MN | 60253±4114 | 6029±865 | 0.77±0.01 | 9.70±0.21 |
|  | HN | 49925±16412 | 8920±3971 | 0.78±0.03 | 10.28±0.96 |
| November | N0 | 61968±5457 | 4039±188 | 0.68±0.01 | 8.20±0.19 |
|  | LN | 59065±1999 | 4508±885 | 0.67±0.03 | 8.21±0.65 |
|  | MN | 66582±2548 | 3239±627 | 0.65±0.02 | 7.66±0.32 |
|  | HN | 65433±2069 | 3117±199 | 0.64±0.01 | 7.51±0.18 |

N0 = 0 g N m^-2^ year^-1^; LN = 1.8 g N m^-2^ year^-1^; MN = 3.6 g N m^-2^ year^-1^; HN = 7.2 g N m^-2^ year^-1^
