# Supplementary material for: Different Responses of Soil Bacterial Communities to Nitrogen Addition in Moss Crust
Source: Front Microbiol. 2021 Sep 10;12:665975. doi: 10.3389/fmicb.2021.665975 (PMC8460773; doi:10.3389/fmicb.2021.665975)
Supplement: Supplementary file 1 [file Data_Sheet_1.zip › Table 2.DOCX]

**Table S2.** Effects of nitrogen addition, season, and their interactions on bacterial community as indicated by two-way ANOVA statistics, based on the relative abundance of genera level.

| Factors | Nitrogen | |  | Season | |  | Nitrogen × Season | |
| --- | --- | --- | --- | --- | --- | --- | --- | --- |
|  | F | P |  | F | P |  | F | P |
| *Sphingomonas* | 4.672 | 0.036 |  | 178.959 | <0.001 |  | 5.331 | 0.003 |
| *Balneimonas* | 11.837 | 0.003 |  | 4.439 | 0.029 |  | 3.027 | 0.036 |
| *Kaistobacter* | 5.470 | 0.024 |  | 394.048 | <0.001 |  | 4.579 | 0.007 |
| *Rubrobacter* | 0.874 | 0.494 |  | 67.031 | <0.001 |  | 2.651 | 0.120 |
| *Geodermatophilus* | 0.763 | 0.546 |  | 27.113 | <0.001 |  | 3.288 | 0.027 |
| *Afifella* | 0.813 | 0.521 |  | 37.793 | <0.001 |  | 1.581 | 0.235 |
| *Massilia* | 1.238 | 0.358 |  | 22.888 | 0.001 |  | 1.388 | 0.295 |
| *Rhodocytophaga* | 0.531 | 0.673 |  | 20.923 | <0.001 |  | 1.971 | 0.130 |
| *Adhaeribacter* | 1.861 | 0.215 |  | 89.396 | <0.001 |  | 2.496 | 0.067 |
| *Flavisolibacter* | 3.040 | 0.093 |  | 8.357 | 0.003 |  | 1.831 | 0.220 |
