# Supplementary material for: Different Responses of Soil Bacterial Communities to Nitrogen Addition in Moss Crust
Source: Front Microbiol. 2021 Sep 10;12:665975. doi: 10.3389/fmicb.2021.665975 (PMC8460773; doi:10.3389/fmicb.2021.665975)
Supplement: Supplementary file 1 [file Data_Sheet_1.zip › Table 3.DOCX]

**Table S3.** The relationships between the soil bacterial diversity (OTU richness, Pielou index, Shannon index) and soil environment factors.

| Soil factor | OTU richness | Pielou index | Shannon index |
| --- | --- | --- | --- |
| pH | 0.384* | 0.519** | 0.504** |
| AN | -0.189 | -0.238 | -0.220 |
| TN | -0.184 | -0.225 | -0.211 |
| AP | -0.149 | -0.293 | -0.268 |
| TP | -0.121 | -0.241 | -0.225 |
| AK | -0.397* | -0.198 | -0.274 |
| SOC | -0.594*** | -0.553*** | -0.597*** |
| NO_3_^-^-N | -0.031 | -0.005 | -0.025 |
| Soil moisture | -0.559*** | -0.915*** | -0.851*** |

*p < 0.01, **p < 0.01, ***p < 0.001.
