# Supplementary material for: Different Responses of Soil Bacterial Communities to Nitrogen Addition in Moss Crust
Source: Front Microbiol. 2021 Sep 10;12:665975. doi: 10.3389/fmicb.2021.665975 (PMC8460773; doi:10.3389/fmicb.2021.665975)
Supplement: Supplementary file 1 [file Data_Sheet_1.zip › Table 4.DOCX]

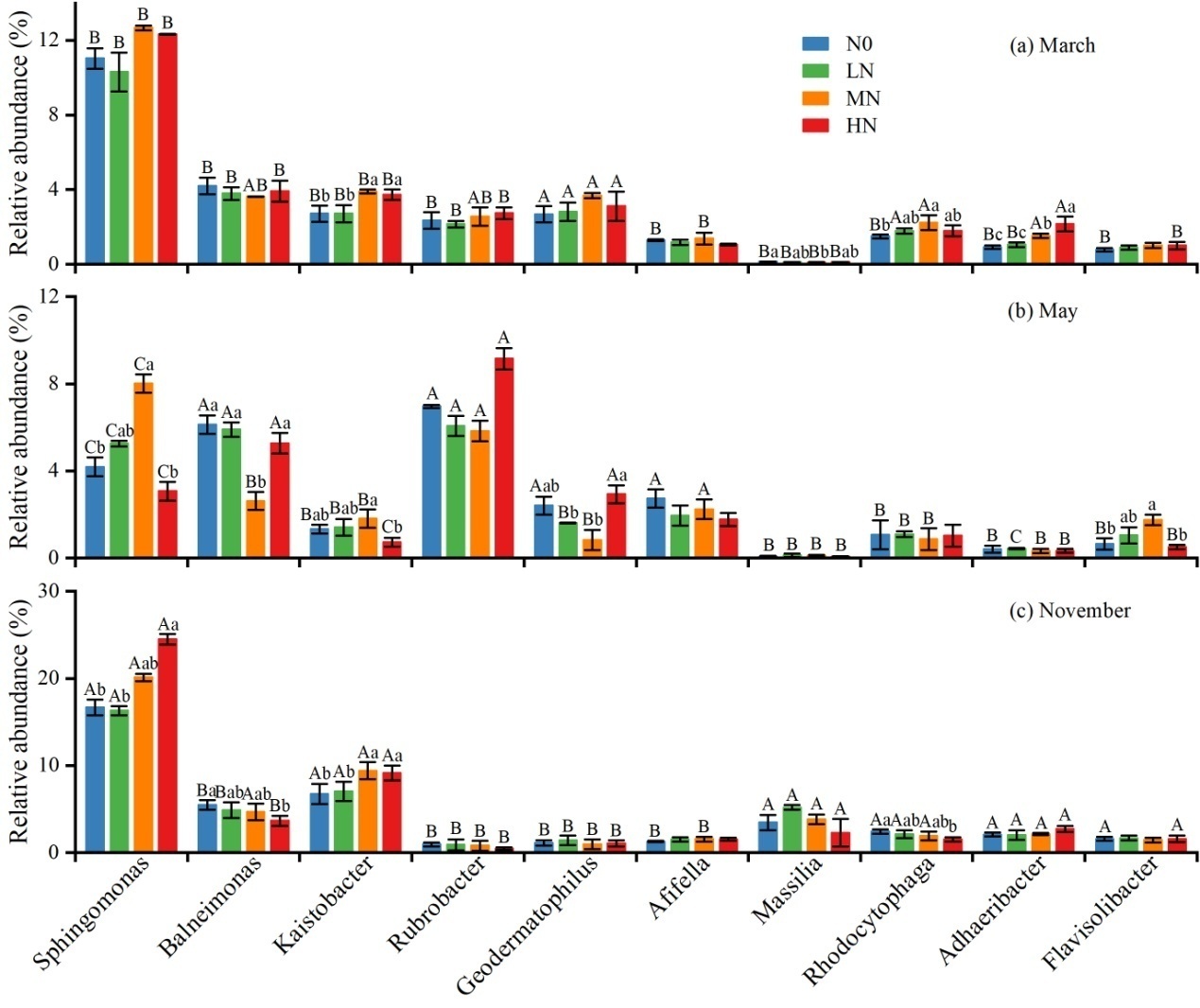


**Figure S1.**The average relative abundance (%) of bacterial taxa at the genus level (n = 3). N0 = 0 g N m^-2^ year^-1^; LN = 1.8 g N m^-2^ year^-1^; MN = 3.6 g N m^-2^ year^-1^; HN = 7.2 g N m^-2^ year^-1^. Different capital letters indicate a significant difference in relative abundance among three sampling months; different lowercase letters indicate a significant difference among four N treatments in the same sampling month. Vertical bars show the standard error (SE) (n = 3).
